# Cost minimization analysis of different growth hormone pen devices based on time-and-motion simulations

**DOI:** 10.1186/1472-6955-9-6

**Published:** 2010-04-08

**Authors:** Nancy A Nickman, Sandra W Haak, Jaewhan Kim

**Affiliations:** 1 Pharmacotherapy Outcomes Research Center, University of Utah, 421 Wakara Way Suite 208, Salt Lake City, Utah 84108, USA; 2 College of Nursing, University of Utah, 10 South 2000 East, Salt Lake City, Utah 84112-5880, USA; 3 Department of Family and Preventive Medicine, School of Medicine, University of Utah; 375 Chipeta Way, Suite A, Salt Lake City, Utah 84108, USA

## Abstract

**Background:**

Numerous pen devices are available to administer recombinant Human Growth Hormone (rhGH), and both patients and health plans have varying issues to consider when selecting a particular product and device for daily use. Therefore, the present study utilized multi-dimensional product analysis to assess potential time involvement, required weekly administration steps, and utilization costs relative to daily rhGH administration.

**Methods:**

Study objectives were to conduct 1) Time-and-Motion (TM) simulations in a randomized block design that allowed time and steps comparisons related to rhGH preparation, administration and storage, and 2) a Cost Minimization Analysis (CMA) relative to opportunity and supply costs. Nurses naïve to rhGH administration and devices were recruited to evaluate four rhGH pen devices (2 in liquid form, 2 requiring reconstitution) via TM simulations. Five videotaped and timed trials for each product were evaluated based on: 1) Learning (initial use instructions), 2) Preparation (arrange device for use), 3) Administration (actual simulation manikin injection), and 4) Storage (maintain product viability between doses), in addition to assessment of steps required for weekly use. The CMA applied micro-costing techniques related to opportunity costs for caregivers (categorized as wages), non-drug medical supplies, and drug product costs.

**Results:**

Norditropin^® ^NordiFlex and Norditropin^® ^NordiPen (NNF and NNP, Novo Nordisk, Inc., Bagsværd, Denmark) took less weekly Total Time (p < 0.05) to use than either of the comparator products, Genotropin^® ^Pen (GTP, Pfizer, Inc, New York, New York) or HumatroPen^® ^(HTP, Eli Lilly and Company, Indianapolis, Indiana). Time savings were directly related to differences in new package Preparation times (NNF (1.35 minutes), NNP (2.48 minutes) GTP (4.11 minutes), HTP (8.64 minutes), p < 0.05)). Administration and Storage times were not statistically different. NNF (15.8 minutes) and NNP (16.2 minutes) also took less time to Learn than HTP (24.0 minutes) and GTP (26.0 minutes), p < 0.05). The number of weekly required administration steps was also least with NNF and NNP. Opportunity cost savings were greater in devices that were easier to prepare for use; GTP represented an 11.8% drug product savings over NNF, NNP and HTP at time of study. Overall supply costs represented <1% of drug costs for all devices.

**Conclusions:**

Time-and-motion simulation data used to support a micro-cost analysis demonstrated that the pen device with the greater time demand has highest net costs.

## Background

Injectable human growth hormone (hGH) has been in use since the late 1950s for treatment of pediatric and adult patients with growth hormone deficiency [1,2]. Traditionally, hGH formulations required reconstitution of lyophilized powder and transfer from vial to syringe prior to intramuscular administration. The first recombinant somatropin (somatropin [rDNA origin], or rhGH) was approved in 1985 by the United States (US) Food and Drug Administration,^2 ^and liquid formulations of rhGH (Nutropin AQ^® ^and Norditropin SimpleXx^®^) became available in the mid-late 1990s [3-5]. Largely due to pediatric use of rhGH and potential fear of syringes and needles in this population [1], manufacturers began to improve subcutaneous administration methods via administration device development.

Similar to patients diagnosed with Type 1 diabetes who require daily insulin replacement, rhGH must also be administered daily in order to achieve maximum therapeutic effect. Therefore, application of insulin injection devices to rhGH administration was pursued [6]. The first insulin pen injector, NovoPen,^® ^was introduced in 1985, with subsequent development and release of the prefilled, disposable FlexPen^® ^insulin device [7]. Liquid formulation of rhGH in the United States initially consisted of a liquid-based product in a vial (Nutropin AQ^® ^10 mg vials) but eventually a Nutropin AQ^® ^pen device with liquid formulation of rhGH was introduced to the US market. Other pen devices were developed, including application of the insulin-based NovoPen^® ^and FlexPen^® ^technologies to rhGH administration with the Norditropin NordiPen^® ^(pre-mixed, pre-filled rhGH cartridges) and Norditropin NordiFlex^® ^(pre-mixed, pre-filled disposable rhGH injection device) [8].

Published patient preference studies of insulin injection devices have also guided development of rhGH administration injection devices [9,10]. Recent evaluations of patient preferences for rhGH injection devices indicated that patients valued "ease of use" [11] and device characteristics such as "reliability, ease of use, lack of pain during injection, safety on use and in storage, and the number of steps in preparation before use, during use and after" [12] as the most important attributes when selecting a device for rhGH replacement therapy.

Substitution of one product for another where more than one product choice is available may not always be useful or recommended for biologically engineered (biosimilar) medications [13]. In addition, product price alone is not always the best discriminator with regard to pharmacy formulary decision-making, and pharmacy benefit managers are creating new ways of managing costs relative to specialty pharmaceutical products [14]. Although evidence-based clinical guidelines should be the main consideration when selecting treatment options, a product's "ease of use" characteristics should also be considered. Ease of use characteristics become especially important with regard to rhGH, which must be either parent-administered (in the case of small children) or self-administered once a day in the evening, often for several years or more. Medication compliance may also be impacted relative to preparation, administration and ease of use characteristics [11,12]. For patients (and parents of patients) learning to safely administer rhGH, product ease of use characteristics also become important in allowing training to take place in as short a period of time as possible. Therefore, the present study was conducted in order to evaluate these characteristics relative to four locally available rhGH products.

## Methods

The present study evaluated the number of steps, potential time involvement relative to learning to administer rhGH, and utilization costs (opportunity costs, non-drug supply costs and drug costs) of daily rhGH administration via four rhGH pen devices listed in Table 1. At the time of the study, Norditropin NordiFlex^® ^(NNF, Novo Nordisk, Inc., Bagsværd, Denmark) [8] was the only pre-filled, pre-mixed disposable pen available. The Norditropin NordiPen^® ^(NNP, Novo Nordisk, Inc., Bagsværd, Denmark) [8] was also available as a ready-to-administer pre-filled liquid cartridge, and neither product required reconstitution prior to use. The Genotropin^® ^Pen (GTP, Pfizer, Inc, New York, New York) [15] and HumatroPen^® ^(HTP, Eli Lilly and Company, Indianapolis, Indiana) [16] pen devices were selected for comparison based on local availability at the time of the study and because reconstitution was required (Table 2). Prior to the study, the Norditropin NordiFlex^® ^pen was believed to be faster to use and easier to prepare for administration because of the pre-mixed, pre-filled, disposable nature of the device.

**Table 1:** Evaluated Products and Devices

Drug & Device	Manufacturer	Product & Device Description
Norditropin NordiFlex^®^ (NNF) 5 mg	Novo Nordisk	Disposable, dial-a-dose, pre-filled injection pen; delivers doses from 0.025 to 1.5 mg in increments of 0.025 mg http://www.norditropin-us.com/parents/nordiflex.asp

Norditropin NordiPen^®^ (NNP) 5 mg CRT	Novo Nordisk	Disposable, pre-filled cartridges to be inserted into re-usable NordiPen dial-a-dose injection pens http://www.norditropin-us.com/parents/nordipen.asp

Genotropin^® ^Pen (GTP) 5.8 mg PFS CRT	Pfizer	Intra-Mix 2-chamber cartridge attached to Genotropin^® ^Pen 5 http://www.genotropin.com/content/Resources_video_guide_ch.aspx

HumatroPen^®^ (HTP) 6 mg CRT	Eli Lilly	Cartridge reconstitution kit (lyophilized powder in cartridge with separate diluent in pre-filled diluent syringe); reconstituted cartridge attached to HumatroPen^® ^ http://www.humatrope.com/common_pages/flash/index.html

**Table 2:** Cost and Product Reconstitution Method by Device

Drug & Device	WAC^a ^$/mg	Reconstitution Method	Final Concentration	Injection Amount
**NNF**	$52.65	Provided in liquid form	5 mg/1.5 ml	Dial to 0.6 mg on device

**NNP**	$52.65	Provided in liquid form	5 mg/1.5 ml	Dial to 0.6 mg on device

**GTP**	$46.42	Two-chamber cartridge	5 mg/ml	Dial to 0.6 mg on device

**HTP**	$52.65	Infuse powder in cartridge with diluent from pre-filled syringe	2.08 mg/ml	Dial to 0.6 mg on device

^a^US Wholesale Acquisition Cost (WAC) as of February 2009

**Figure 1:**
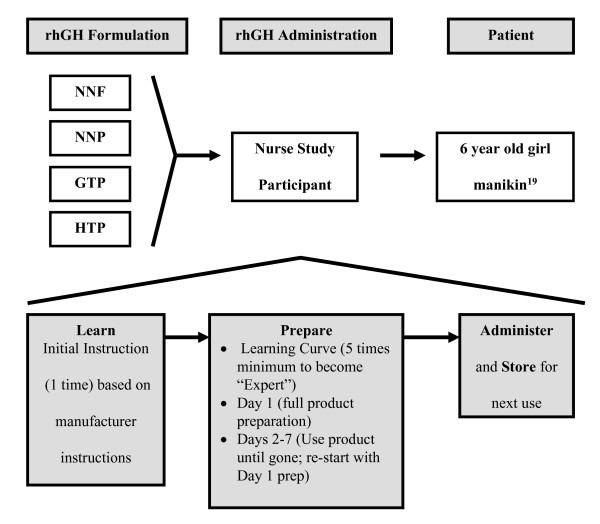
**Simulation scenario based on administration of recombinant Human Growth Hormone (rhGH) to simulated 50^th ^percentile weight 6 year old girl**.

A simulated Time-and-Motion (TM) analysis was designed based on standard industrial engineering techniques [17,18] in order to determine the potential amount of patient/parent time required to Learn (initial instructions for use), Prepare (arrange device for use), Administer the dose to a simulated child [19] (actual injection), and Store (provide for product viability between doses) each of the four devices and assess the complexity involved with the use of each device (Figure 1). Although the ideal study would have evaluated actual patient and/or parental learning relative to each device, nurses were recruited to participate in the study as described below.

The University of Utah College of Nursing Learning Resource Center (LRC) was utilized for the TM simulations, because the laboratory possesses state-of-the-art practice simulation areas that are used to train students. Six registered nurses who had prior experience giving subcutaneous injections but were naïve to rhGH administration and the pen injection devices were recruited by word of mouth. The simulation room setup consisted of:

• a Learning station with a computer and print materials pertinent to all of the devices under study;

• a medication Preparation station with all devices and supplies necessary for medication preparation and administration;

• an Administration station with a child size manikin [19] for actual medication administration lying on a hospital bed; and

• a Storage station consisting of a table for medication to be set upon ("stored") between first and second simulation doses.

The distance from the Learning station to the other stations was approximately 6 feet, with only 4 steps required to move between each of the other stations. The order of pen device simulations was randomized by product across participants (randomized block design), so that bias against one device based on learning accrued from using another device was minimized.

At the beginning of each set of pen device simulation trials, participants were given manufacturer instructional materials that were intended for use by patients and families (see Table 1). Instructional materials for NNF consisted of an instructional DVD provided as part of a patient starter kit. For NNP, GTP and HTP, instructional videos were available via the internet. Learning materials were only reviewed once by participants prior to proceeding to the medication Preparation station, although instructional materials remained available in case of additional questions.

Each trial consisted of first dose (Dose 1) preparation of the pen device including reconstitution if required, administration of a dose to the manikin, and storage of the device. This was followed by a second dose (Dose 2) using the pen device prepared as part of the Dose 1 simulation. Participants retrieved the pen device from the Storage station, brought it to the Preparation station, attached a new needle to the pen, and administered Dose 2 to the manikin. Because activities related to preparation and administration of Dose 2 were assumed to be the same once Dose 1 preparation was completed, all simulations were conducted and analyzed based on the steps a parent would complete in order to administer Dose 1 and Dose 2 of each pen device. Dose 2 was also conceived as being identical in steps and character to preparation, administration, and storage methods that would be used by a parent until preparation of a new pen device would become necessary. Then Dose 1 and subsequent doses would be administered from the new pen device. Five iterations of each Dose 1 and Dose 2 simulation were completed for each pen device in a randomized block design, resulting in a total of 60 observations (30 observations each Dose 1 and Dose 2) across the six simulation participants.

Dosages used during simulations were based on a 4.2 mg/week average dose [2], resulting in a 0.6 mg daily dose for a 6 year old girl weighing 20 kg (50^th ^percentile weight based on US standards) [20]. Non-drug (medical) supplies required as part of the administration process were also quantified. All simulation sessions were videotaped and timed; analysis was completed by project staff. Statistical analysis was completed using descriptive statistics at an *a priori *significance level of 0.05. Since the primary purpose of the evaluation was to compare use of NNF relative to NNP, GTP and HTP, all time comparisons were completed relative to the NNF pen device and evaluated using one-way analysis of variance (ANOVA) for repeated measures design. A Tukey test *post hoc *analysis of results was conducted to determine where time differences occurred if present. Tapes and manufacturer supplied educational materials were also assessed in order to count the number of steps required to Prepare, Administer, and Store each pen device.

A Cost Minimization Analysis (CMA) using micro-cost methods [21,22] was conducted to determine potential time and supplies cost savings through the use of one pen device over another. All US minimum wage labor cost information [23] and Wholesale Acquisition Cost (WAC) price information for each of the four rhGH drug products [24] was collected from a nationally available database, while non-drug medical supply costs (needles, alcohol sponges, dry sponges, and bandages) were derived from a nationally available nursing supplies catalog [25]. The entire study was classified as "Minimal Risk" by the University of Utah Institutional Review Board, and all nurse participants signed Informed Consent forms to that effect.

## Results and Discussion

### Time-and-Motion (TM) Simulation Results

The order of pen device simulations and evaluation of products listed in Table 1 was conducted in a randomized block design in order to minimize learning transfer from one device to the next. Each of 6 participants completed 5 simulations for the 4 pen devices resulting in a total number of 30 observations each for first (Dose 1) and second (Dose 2) doses of each pen device (total of 60 observations per pen device). As outlined in Figure 1 and based on learning theory [26], participants were estimated to become "experts" in using the devices after preparing and administering two doses 5 times each. Four variables were assessed (Learning, Preparation, Administration, and Storage) as part of a multi-dimensional analysis of the rhGH pen devices.

The Learning variable was assessed based on the length of time taken by participants to review manufacturer provided DVD and internet-based video materials one time, and results are listed in Table 3. The amount of time required to learn to use the NNF, NNP and HTP devices were statistically different from GTP (p < 0.05). Learning variable time results ranged from a low of 7 minutes (NNP manufacturer internet instructions) to a high of 33 minutes (HTP manufacturer internet instructions), although average Learning times ranged from 15.8 minutes (NNF) to an average of 26.0 minutes (GTP).

**Table 3:** Time Differences in Learning to Use Devices

Drug & Device	Learning Time (mins)		
	**Average (s.d.)**	**Range**	**95% C.I**.

**NNF**	15.83 (2.86)	12.00 - 21.00	12.83 - 18.83

**NNP**	16.17 (8.35)	7.00 - 28.00	7.40 - 24.93

**GTP**	26.00* (4.77)	19.00 - 32.00	20.99 - 31.01

**HTP**	24.00 (8.29)	15.00 - 33.00	15.30 - 32.71

*p < 0.05 relative to NNF; N = 6 trials

The remaining results from Preparation, Administration, and Storage analysis of the TM simulations are listed in Additional File 1, Table S1. In terms of average Dose 1 Preparation times, NNP (2.48 minutes) GTP (4.11 minutes), and HTP (8.64 minutes) were all significantly different from NNF (1.35 minutes, p < 0.05). With regard to GTP and HTP which both require reconstitution prior to use, the results were not surprising. The Dose 1 Preparation time differences between NNF and NNP are likely due to the additional NNP step requirement to insert an already liquefied rhGH cartridge into the NNP pen device, a step not required in the ready-to-use disposable NNF pen device. Dose 2 preparation times were noticeably faster than Dose 1 preparation times, with only average GTP time (1.30 minutes) evaluated as significantly different than NNF (0.86 minutes), NNP (0.92 minutes), and HTP (0.94 minutes) average Dose 2 preparation times. Once prepared for use, no significant differences were seen in Administration time between the pen devices (all device Dose 1 ranges 0.63-0.68 minutes; all device Dose 2 ranges 0.56-0.63 minutes). Only GTP was significantly different (p < 0.05) with regard to Storage, regardless of whether either Dose 1 or Dose 2 were being stored for the next administration.

Overall and on average, NNF (Dose 1 = 2.33 minutes; Dose 2 = 1.75 minutes) and NNP (Dose 1 = 3.61 minutes, p < 0.05, Dose 2 = 1.94 minutes) took less Total Time than HTP (Dose 1 = 9.77 minutes, p < 0.05, Dose 2 = 1.95 minutes) and GTP (Dose 1 = 5.46 minutes, p < 0.05, Dose 2 = 2.45 minutes, p < 0.05) primarily due to differences in Preparation time (p < 0.05). Users became more efficient in performing each step with practice (i.e., took less time to perform) and individual Total Times decreased for each participant with each succeeding trial of each pen device (p < 0.05).

**Figure 2:**
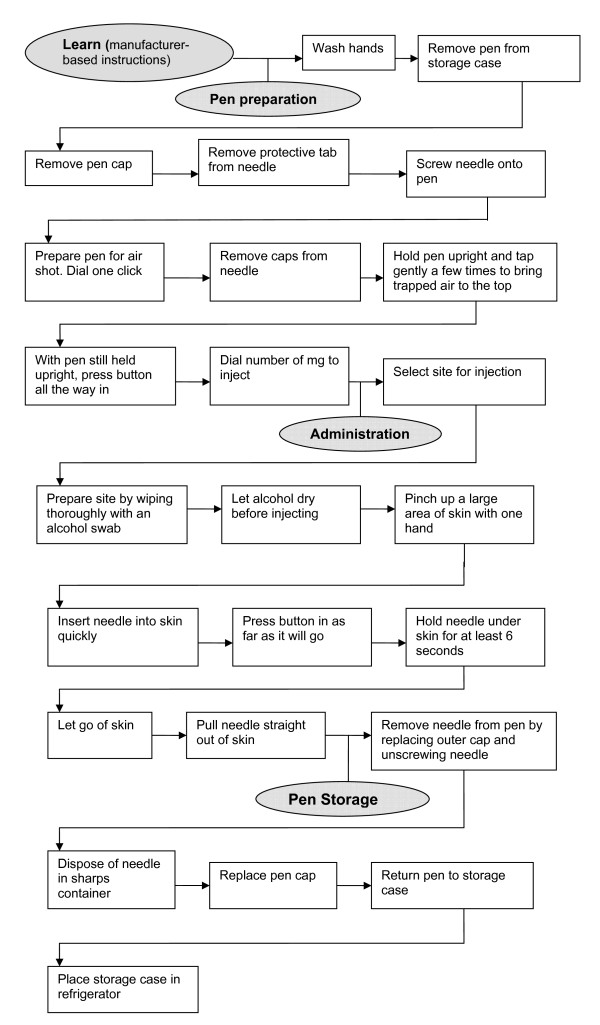
**Process flow diagram of common manufacturer instructions and time-and-motion simulation steps associated with use of studied recombinant Human Growth Hormone (rhGH) pens**.

**Figure 3:**
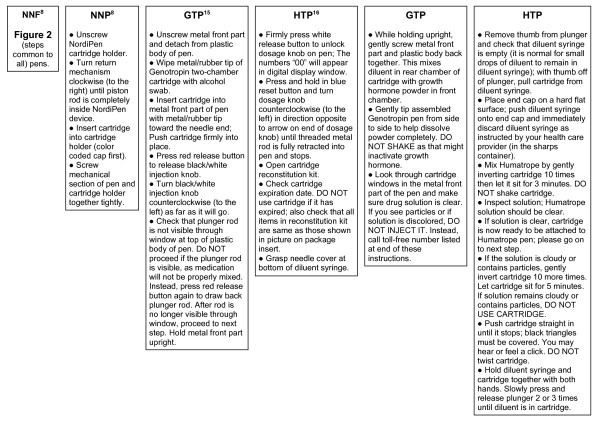
**Pen-specific additional manufacturer instructions and preparation steps required for use beyond those listed in Figure 2**.

Process flow diagrams (Figures 2 and 3) were created based on manufacturer supplied patient instructions for each pen device. Figure 2 outlines manufacturer suggested usage steps common to all pens, while Figure 3 outlines additional manufacturer suggested preparation steps that were required of the NNP, GTP, and HTP pens beyond that of the NNF pen. Of note, only the manufacturer instructions for HTP [16] asked users to check an expiration date, although best practices dictate checking expiration dates of all medicines.

Differences between pens identified in both the process flow charts and the TM analysis results were also reinforced in the results of the Step Analysis (Additional File 1, Table S2). The combined number of steps outlined in Figure 2 and Figure 3 process flow charts indicate that the number of preparation steps for GTP (20 steps) and HTP (27 steps) were nearly double those required by NNF (11 steps) and NNP (15 steps). Although the total number of steps that must be completed on a weekly basis in order to administer a daily dose are not dramatically different (low of 175 steps for NNF and 179 steps for NNP to high of 185 steps for GTP and 186 steps for HTP), the NNF and NNP devices require less preparation manipulation due to the availability for users of rhGH in a liquid, ready-to-use form. Even the small number of required additional preparation steps necessary for use of GTP and HTP are relatively time consuming (p < 0.05 relative to NNF).

### Cost Minimization Analysis (CMA) Results

CMA results from the micro-cost analysis are listed in Additional File 1, Table S3. The estimated weekly Total Times that resulted from the TM portion of the multi-dimensional analysis were used to estimate required potential patient/parental time necessary for daily rhGH administration. Weekly times were then valued as opportunity costs [22] and valued using a United States minimum wage value of $6.55 per hour [23]. Minimum wage versus other wage estimates was utilized given that such relatively small time requirements for daily and weekly administration would not require a parent or young adult to miss work in order to administer to either a child or self, and it is highly unlikely that skilled care providers would be brought into the home for daily rhGH administration. Opportunity cost savings, albeit small at $1,100 or less per year, were greatest with those pen devices that were easier to prepare for use, especially when multiple doses, and hence preparatory activities, must be made on a weekly basis. Weekly non-drug medical supply costs (needles, alcohol sponges, dry sponges, and bandages) represented less than 1% of drug costs for all devices. Although not insignificant on a yearly basis, cost differences between required supplies for the 4 comparator pen devices were relatively small. WAC price estimates used to calculate the opportunity cost savings relative to selection of one product over another were only different for GTP, representing an 11.8% opportunity cost savings over NNF, NNP and HTP at the time of this study. Since contracting discounts and tiered pricing strategies might be utilized by benefit managers in actual practice, drug price comparisons should be performed on an individual basis to assess potential savings [13,14].

Previous research has documented that patients desire rhGH administration devices that are easy to use, require less steps for successful administration, and provide the opportunity for improved compliance with daily therapy [12]. Benefit managers are also recognizing that improved clinical outcomes may be related in some measure to medication administration methods [14], leading to the consideration of pen devices such as those evaluated in this study. In addition, device function factors such as number of steps required for administration that potentially impact safe and accurate use should also be considered [27]. Devices that require fewer steps may lead to reductions in dosing errors. Although safety per se was not evaluated in the present study, device and medication regimen factors such as simplicity should be considered when primary usage is geared toward lay population users [12,28-30]. In order for parents to administer rhGH in the home, patients/parents need to learn to prepare, administer and store rhGH, in addition to learning to perform injections in an accurate and reliable manner. If devices are easier for patients and caregivers to learn and use, it may also be easier for them to be adherent to medication regimens.

### Limitations

Nurses served as consenting participants in this study. Although participants were not representative of an "average" patient or parent who is likely to be unfamiliar with injection administration techniques, nurse participants were unfamiliar with rhGH administration in general, and naïve to the four comparator pen devices. In addition, only 6 nurses participated in the study. Statistical power at the 0.05 level required a minimum of 30 observations per device, and the study was designed to maximize observations (30 observations each for Dose 1 and Dose 2) while minimizing purchase and wastage relative to study product costs.

Finally, micro-cost and cost minimization analyses are dependent upon local and regional cost variations for drugs and non-drug medical supplies, which may also differ from global costs. However, analyses modelling formulae allow for cost substitutions based upon local rates.

## Conclusions

NNF and NNP took less Total Time vs. GTP and HTP due to a shorter amount of time needed to learn and prepare rhGH for daily administration. Time-and-motion simulation data used to support a micro-cost analysis also demonstrated the value of multi-dimensional product-device analysis.

## Competing interests

This study was supported by Novo Nordisk, Inc., Princeton, NJ. Authors Nickman, Haak and Kim have no financial or non-financial competing interests in the study, analysis or manuscript.

## Authors' contributions

NAN and SWH conceptualized, designed, carried out, and analyzed the plan of study, results, presentations and manuscript from the current study. JWK participated in conceptualization of statistical and economic analysis and participated in writing of the manuscript. Each author also read and approved the final manuscript.

## Pre-publication history

The pre-publication history for this paper can be accessed here:

http://www.biomedcentral.com/1472-6955/9/6/prepub

## Supplementary Material

Additional file 1 **Additional File **1, **Table S1 - Time Differences in Weekly Preparation, Administration & Storage**. Results of time-and-motion analysis for Preparation, Administration & Storage variables Click here for file

Additional file 2 **Additional File **1, **Table S2 - Step Differences in Weekly Preparation, Administration & Storage**. Results of step analysis for Preparation, Administration & Storage variables Click here for file

Additional file 3 **Additional File **1, **Table S3 - Opportunity and Supplies Cost Differences in Weekly and Yearly Preparation, Administration & Storage**. Results of time and supplies opportunity cost analysis for Preparation, Administration & Storage variables on a weekly and yearly basis Click here for file
