# Supplementary material for: Cost minimization analysis of different growth hormone pen devices based on time-and-motion simulations
Source: BMC Nurs. 2010 Apr 8;9:6. doi: 10.1186/1472-6955-9-6 (PMC2858139; doi:10.1186/1472-6955-9-6)
Supplement: Additional file 1 — , Table S1 - Time Differences in Weekly Preparation, Administration & Storage. Results of time-and-motion analysis for Preparation, Administration & Storage variables [file 1472-6955-9-6-S1.DOC]

## Additional File 1, Table S1 - Time Differences in Weekly Preparation, Administration & Storage

| **Drug & Device** | **Dose 1** | | | **Dose 2** | | |
| --- | --- | --- | --- | --- | --- | --- |
|  | **Average (s.d.)** | **Range** | **95% C.I.** | **. Average (s.d.)** | **Range** | **95% C.I.** |
|  | **Preparation Time (mins)** | | | | | |
| **NNF** | 1.35 (0.51) | 0.58 - 2.63 | 1.1 6- 1.54 | 0.86 (0.20) | 0.45 - 1.23 | 0.78 - 0.94 |
| **NNP** | 2.48* (1.61) | 1.28 - 7.75 | 1.87 - 3.08 | 0.92 (0.42) | 0.42 - 2.70 | 0.77 - 1.08 |
| **GTP** | 4.11* (1.67) | 2.17 - 8.45 | 3.49 - 4.73 | 1.30* (0.49) | 0.67 - 2.23 | 1.12 - 1.49 |
| **HTP** | 8.64* (3.37) | 5.70 - 8.45 | 7.38 - 9.90 | 0.94 (0.56) | 0.50- 3.07 | 0.73 - 1.14 |
|  | **Administration Time (mins)** | | | | | |
| **NNF** | 0.63 (0.19) | 0.40 - 1.13 | 0.56 - 0.70 | 0.56 (0.20) | 0.23 - 0.97 | 0.49 -0.64 |
| **NNP** | 0.63 (0.17) | 0.35 - 1.13 | 0.57 - 0.70 | 0.57 (0.14) | 0.28 - 0.83 | 0.51 - 0.62 |
| **GTP** | 0.67 (0.21) | 0.40 - 1.17 | 0.60 - 0.75 | 0.56 (0.17) | 0.30 - 0.82 | 0.50 - 0.62 |
| **HTP** | 0.68 (0.26) | 0.38 - 1.30 | 0.58 - 0.77 | 0.63 (0.24) | 0.33 - 1.35 | 0.55 - 0.72 |
|  | **Storage Time (mins)** | | | | | |
| **NNF** | 0.35 (0.12) | 0.17 - 0.67 | 0.31 - 0.40 | 0.33 (0.09) | 0.18 - 0.52 | 0.30 - 0.37 |
| **NNP** | 0.50 (0.45) | 0.18 - 2.45 | 0.33 - 0.67 | 0.45 (0.35) | 0.20 - 2.00 | 0.32 - 0.58 |
| **GTP** | 0.68* (0.39) | 0.32 - 2.05 | 0.53 - 0.83 | 0.59* (0.18) | 0.35 - 1.08 | 0.52 - 0.65 |
| **HTP** | 0.45 (0.23) | 0.22- 1.13 | 0.36 - 0.53 | 0.38 (0.13) | 0.23 - 0.77 | 0.33 - 0.43 |
|  | **Total Time (mins)** | | | | | |
| **NNF** | 2.33 (0.66) | 1.31 - 3.85 | 2.08 - 2.57 | 1.75 (0.36) | 1.08 - 2.36 | 1.62 - 1.89 |
| **NNP** | 3.61* (2.00) | 2.00 - 10.83 | 2.86 - 4.35 | 1.94 (0.71) | 1.10 - 4.17 | 1.67 - 2.20 |
| **GTP** | 5.46* (2.03) | 2.94 - 10.47 | 4.71 - 6.22 | 2.45* (0.72) | 1.42 - 4.06 | 2.18 - 2.72 |
| **HTP** | 9.77* (3.57) | 6.50 - 22.07 | 8.43 - 11.10 | 1.95 (0.75) | 1.08 - 4.13 | 1.67 - 2.23 |
| *p<0.05 relative to NNF; N=30 trials each Dose 1 and Dose 2  **Assumes daily administration, preparation of 1 package of product/week and that second/consecutive doses consume equal time | | | | | | |
