# Supplementary material for: Cost minimization analysis of different growth hormone pen devices based on time-and-motion simulations
Source: BMC Nurs. 2010 Apr 8;9:6. doi: 10.1186/1472-6955-9-6 (PMC2858139; doi:10.1186/1472-6955-9-6)
Supplement: Additional file 2 — Additional File 1, Table S2 - Step Differences in Weekly Preparation, Administration & Storage. Results of step analysis for Preparation, Administration & Storage variables [file 1472-6955-9-6-S2.DOC]

## Additional File 1, Table S2 - Step Differences in Weekly Preparation, Administration & Storage

| **Drug & Device** | **Preparation Steps**  **Dose 1 (N)** | **Preparation Steps**  **Dose 2 (N)** | **Administration Steps**  **(N)** | **Storage Steps**  **(N)** | ***Weekly Total Steps**  **(N)** |
| --- | --- | --- | --- | --- | --- |
| **NNF** | 11 | 11 | 9 | 5 | **175** |
| **NNP** | 15 | 11 | 9 | 5 | **179** |
| **GTP** | 20 | 10 | 9 | 6 | **185** |
| **HTP** | 27 | 9 | 9 | 6 | **186** |
| *****Weekly Total Steps = (Dose 1 steps x 1) + (Dose 2 steps x 6) | | | | | |
