# Supplementary material for: Cost minimization analysis of different growth hormone pen devices based on time-and-motion simulations
Source: BMC Nurs. 2010 Apr 8;9:6. doi: 10.1186/1472-6955-9-6 (PMC2858139; doi:10.1186/1472-6955-9-6)
Supplement: Additional file 3 — Additional File 1, Table S3 - Opportunity and Supplies Cost Differences in Weekly and Yearly Preparation, Administration & Storage. Results of time and supplies opportunity cost analysis for Preparation, Administration & Storage variables on a weekly and yearly basis [file 1472-6955-9-6-S3.DOC]

## Additional File 1, Table S3 - Opportunity and Supplies Cost Differences in Weekly and Yearly Preparation, Administration & Storage

|  | **Total Time**  **Average (minutes)** | | **Total Time**  **Cost ($MinWage^c^)** | | **Total Supplies**  **Cost ($)** | | **Total Dose**  **Cost ($)** | |
| --- | --- | --- | --- | --- | --- | --- | --- | --- |
| **Drug & Device** | **Weekly^b^** | **Yearly** | **Weekly^b^** | **Yearly** | **Weekly^b^** | **Yearly** | **Weekly^d^** | **Yearly^d^** |
| **NNF** | 12.83 | 667.16 | $1.40 | $72.80 | $2.11 | $109.72 | $221.15 | $11,499.80 |
| **NNP** | 15.25 | 793.00 | $1.66 | $86.32 | $2.11 | $109.72 | $221.15 | $11,499.80 |
| **GTP** | 20.16^a^ | 1048.32 | $2.20 | $114.40 | $2.15 | $111.80 | $194.97 | $10,138.44 |
| **HTP** | 21.47^a^ | 1116.44 | $2.34 | $121.68 | $2.15 | $111.80 | $221.15 | $11,499.80 |
| ^a^p<0.05 relative to NNF; N=30 trials each Dose 1 and Dose 2  ^b^Assumes daily administration, preparation of 1 package of product/week; second/consecutive doses consume equal time  ^c^US Minimum Wage ($MinWage) = $6.55  ^d^US Wholesale Acquisition Cost (WAC) as of February 2009 | | | | | | | | |
